# Ring-Opening Polymerization of l-Lactic Acid *O*-Carboxyanhydrides Initiated by Alkoxy Rare Earth Compounds

**DOI:** 10.3390/molecules181012768

**Published:** 2013-10-15

**Authors:** Zhengguo He, Lin Jiang, Yongming Chuan, Hongli Li, Minglong Yuan

**Affiliations:** Engineering Research Center of Biopolymer Functional Materials of Yunnan, Yunnan University of Nationalities, Kunming 650500, China

**Keywords:** l-lactic acid *O*-carboxyanhydride, ring-opening polymerization, triisopropoxyneodymium initiator

## Abstract

The ring-opening polymerization of l-lactic acid *O*-carboxyanhydrides was initiated by triisopropoxyneodymium in toluene-THF mixtures. Typically, high yields and relatively high molecular weight PLAs were obtained within 4 h at 25 °C. The reaction was highly controllable and easy to conduct, and the molecular weight distribution of the PLAs was rather narrow (Mw/Mn = 1.10–1.36). NMR analysis showed that one end of the PLA chain consisted of an isopropoxy group, while the other end of the chain contained a hydroxyl group. Due to their availability and high polymerizability, Lac-OCAs are promising monomers for the preparation of tailored architectures derived from well-defined PLAs.

## 1. Introduction

Over the last several decades, interest in the preparation of biodegradable and biocompatible polymers has increased significantly. Many studies have focused on the synthesis of polylactides and polymers containing natural α-amino acids, which can be degraded into metabolites [1,2]. The synthesis of polylactide is generally based on the ring-opening polymerization of l-lactide [3,4,5,6,7]. Polyester synthesis by ROP is largely limited to monomers that do not contain complex functional groups due to the prevalence of undesirable transesterification reactions, which occur in the presence of alcohols, amines and esters and result in a loss of control over the molecular parameters. Furthermore, copolymers of l-lactic acid and α-amino acids (also known as poly (depsipeptides)) cannot be obtained with this method.

In order to circumvent these limitations, activated equivalents of lactide would be highly desirable. Kricheldorf [8] reported a novel approach to the preparation of homopolymers of lactic acid by the polymerization of l-Lac-OCA using a variety of initiators, such as pyridine, triethylamine, KOtBu, and Ti(O-Bu)_4_. However, number average molecular weights greater than 3,000 g/mol were not obtained, and in fact molecular weights less than 3,000 g/mol are inappropriate for most practical applications. Recently, Bourissou [9] obtained high molecular weight (M_n_ values up to 38,400 g/mol) PLAs in high yield within a few hours using PS and Novozym 435 lipase to initiate the ring-opening polymerization of Lac-OCA. A number of researchers [10,11,12] reported the living ring-opening polymerization of l-Lac-OCA in the presence of neo-Pent-OH/DMAP. The monomer/initiator ratio was varied from 10 to 600, and PLAs with molecular weights up to 60,000 g/mol were obtained. In addition, the degree of polymerization closely matched that of the monomer feed. l-Lac-OCA exhibits significantly higher polymerizability than lactide toward 4-dimethylaminopyridine (DMAP), providing access to PLAs with controlled molecular weights and low polydispersities under mild conditions (typically within a few minutes at room temperature with lacOCA *vs.* a few days at 35 °C with lactides). One major drawback to the use of PLAs is that the resulting PLA chain does not contain any functional groups. Subsequently, Boullay reported that poly(*α*-hydroxyacids) featuring pendant carboxylic acid groups were readily prepared from gluOCA, a *O*-carboxy-anhydride derived from glutamic acid [13]. Cheng reported that polyesters bearing pendant amine groups were readily prepared from 5-(4-(prop-2-yn-1-yloxy)benzyl)-1,3-dioxolane-2,4-dione, an *O*-carboxyanhydride derived from tyrosine [14]. Cheng also reported water-soluble poly(α-hydroxyacids) bearing pendant hydroxyl groups that were prepared via ring-opening polymerization of *O*-benzyl-l-serine carboxyanhydrides followed by the removal of the benzyl group [15].

Alkoxy rare earth compounds are considered efficient initiators for the ring-opening polymerization of lactones [16,17,18,19]. So far, metal-promoted polymerization of *O*-carboxyanhydride has only been scarcely explored. Cheng reported the polymerization of *O*-carboxyanhydride initiated by Cpt−metal complex [20]. Previously, we studied the ring-opening polymerization of lactones by rare earth compounds [21,22,23,24,25]. The high polymerizability of the *O*-carboxyanhydride moiety prompted us to speculate that lacOCA may behave as an activated equivalent of lactide toward alkoxy rare earth compounds. Herein, we report the ring-opening polymerization of l-lactic acid *O*-carboxyanhydrides initiated by alkoxy rare earth compounds.

## 2. Results and Discussion

### 2.1. Polymerization of Lac-OCA

In general, metal alkoxides such as aluminum triisopropoxide (Al(OiPr)_3_) [26,27] and rare earth alkoxides are efficient initiators in the ring-opening polymerization of lactones. In the present study, we investigated the ring-opening polymerization of l-lactic acid *O*-carboxyanhydrides initiated by Al(OiPr)_3_. The polymerizations were conducted in toluene at 25 °C and 60 °C, and the Lac-OCA/initiator molar ratios (M/I) were varied from 50–150. However, polymerization did not occur as an initiator with Al(OiPr)_3_. Therefore, the ring-opening polymerization of l-lactic acid *O*-carboxyanhydride was initiated by triisopropoxyneodymium, and the results are shown in Table 1. As shown in the Table 1, the reaction was controllable and easy to conduct, and the molecular weight distribution of the PLAs was rather narrow (Mw/Mn = 1.10–1.36). The polymerization could be conducted in THF or toluene-THF, but PLAs with relatively low molecular weights were obtained when the polymerizations were conducted in THF. However, relatively high molecular weight PLAs was obtained in toluene-THF (approximately 22,000 g/mol). As shown in Table 1, the conversion of PLA at high Lac-OCA/initiator ratios was lower than that of low Lac-OCA/initiator ratios. However, the molecular weight of the corresponding PLAs was relatively low due to the increased number of active species. In contrast, high molecular weight PLAs were obtained at relatively high Lac-OCA/initiator ratios. When the Lac-OCA/initiator molar ratio was greater than 250, the number of active species was insufficient, and polymerization was not initiated. From Table 1, we can see that when the Lac-OCA/initiator molar ratio was 150 the molecular weight is up to highest and a decreasing trend was shown either higher or lower than 150. It could be the reason that when the Lac-OCA/initiator molar ratio was lower than 150 the active species were not enough and the competitive reaction made the active species decreased at the molar ratio higher than 150.

**Table 1:** Polymerization of Lac-OCA Initiated by Nd(O^i^Pr)_3_.

No.	M/I (molar ratio)	solvent volume (mL)	Mn ^a^ (g·moL^−1^)	Mw/Mn ^a^	Monomer conversion ^b^ (%)
1	27.4:1	12.0	11,100	1.31	99.3
2	50:1	12.0	13,000	1.25	99.8
3	100:1	12.0	13,000	1.18	99.5
4	150:1	12.0	16,000	1.36	99.8
5	200:1	12.0	12,600	1.30	97.3
6	250:1	12.0	6,700	1.12	55.7
7	300:1	12.0	/	/	/
8	150:1	12.0	6,600	1.11	99.4

Experiments 1–7 were conducted in toluene-THF (toluene/THF = 2). Experiment 8 was conducted in THF. Lac-OCA concentration = 1.67 moL/L, M/I: monomer/initiator (molar ratio), temperature = 25 °C, reaction time = 4 h. ^a^: Determined from GPC. ^b^: Determined from ^1^H-NMR spectroscopy.

The effect of the polymerization time on the conversion and molecular weight of the PLAs is shown in Table 2. In general, as the polymerization time increased, the conversion of the monomer also increased. As shown in the Table 2, high yields and high molecular weight PLAs were obtained within 4 h. However, when the polymerization time was increased from 4 h to 8 h, significant changes in the conversion of monomer were not observed. Thus, considering the conversion rate and molecular weight of the product, the optimal polymerization time was 4 h. Table 3 summarizes the results of the polymerization of Lac-OCA at different temperatures. The data revealed that the yield and molecular weight of Lac-OCA increased as the reaction temperature increased from 5 °C to 25 °C. However, when the temperature was greater than 25 °C, the molecular weight of PLA decreased slightly. Thus, the maximum yield and molecular weight of PLA were obtained at 25 °C.

**Table 2:** Effects of the Reaction Time on the Polymerization of Lac-OCA.

No.	Time (h)	Mn ^a^ (g·mol^−1^)	Mw/Mn ^a^	Monomer conversion ^b^ (%)
1	0.25	9200	1.18	78.36
2	0.5	10700	1.29	90.47
3	0.75	10000	1.26	92.21
4	1.0	9700	1.32	95.32
5	2.0	13300	1.16	98.51
6	4.0	12800	1.24	99.08
7	8.0	14200	1.15	99.17

Polymerizations were conducted in toluene-THF (toluene/THF = 2), Lac-OCA concentration = 1.67 mol/L, M/I = 150, temperature = 25 °C. ^a^: Determined from GPC. ^b^: Determined from ^1^H-NMR spectroscopy.

**Table 3:** Effects of Temperature on the Polymerization of Lac-OCA.

*T* (°C)	Mn ^a^ (g·mol^−1^)	Mw/Mn ^a^	Monomer conversion ^b^ (%)
5	12,200	1.34	88.43
25	13,900	1.36	99.87
40	13,000	1.39	99.19
60	10,400	1.28	99.21

Polymerizations were conducted in toluene-THF (toluene/THF = 2). Lac-OCA concentration = 1.67 mol/L, M/I = 150, time = 4 h. ^a^: Determined from GPC. ^b^: Determined from ^1^H-NMR spectroscopy.

### 2.2. NMR Analysis of PLA

**Figure 1:**
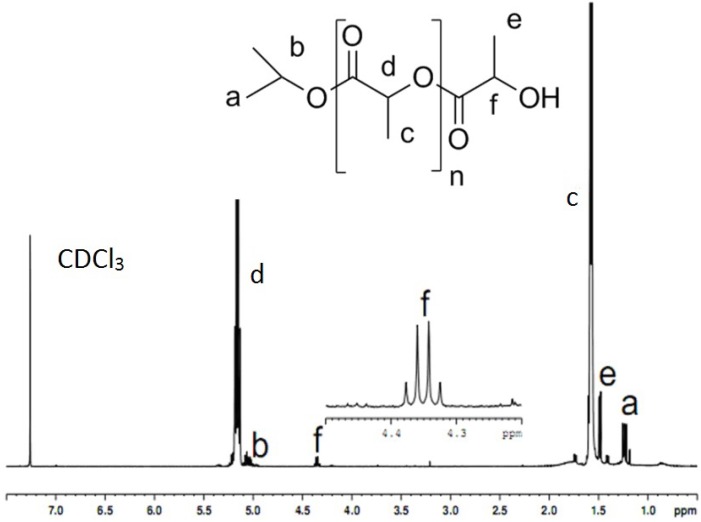
^1^H-NMR spectrum of PLA (the PLA was synthesized in toluene-THF (toluene/THF = 2), Lac-OCA concentration = 1.67 mol/L, M/I = 150, temperature = 25 °C and reaction time = 4 h).

After removing the catalyst/initiator, pure PLA was analyzed by ^1^H-NMR spectroscopy. To observe the polymerization process and the end groups of the PLA chain, the polymer obtained at the lowest M/I ratio, which provided the largest initiator concentration, was analyzed. The diagnostic signals for isotactic PLA, including the methyl proton at 1.6 ppm and the methine proton at 5.20 ppm, were observed in the ^1^H-NMR spectrum. As shown in Figure 1, the isopropyl groups of the initiator were connected to the PLA chain. Namely, the methyl proton of the isopropyl subunit resonated at 1.21 ppm, and the methine proton of the isopropyl groups resonated at 5.01 ppm. The results shown in Figure 1 also indicated that the other end group of the PLA chain contained a hydroxyl group. For instance, the methyl proton at the end of the lactic acid ester moiety resonated at 1.49 ppm, and the methine proton at the end of the lactic acid ester group resonated at 4.33 ppm. The NMR analysis showed that the polymerization proceeded with complete decarboxylation and without epimerization. These results are in accordance with reference 9, which showed that monoesters of carbonic acids resulting from the ring-opening of *O*-carboxyanhydrides are highly unstable with respect to decarboxylation.

**Scheme 1:**
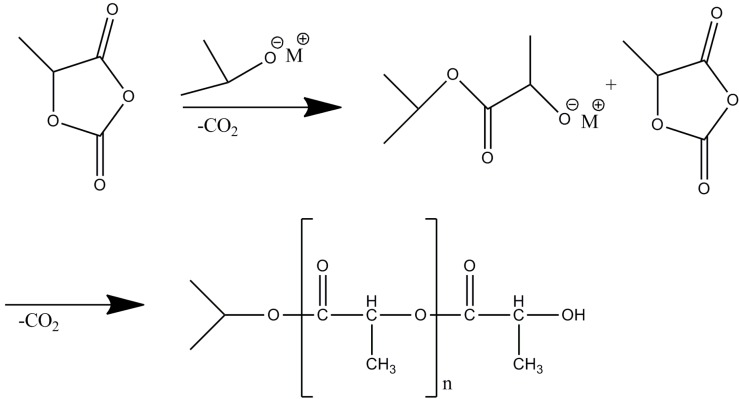
The polymerization of Lac-OCA.

The above results showed that the polymerization process occurred as follows: the negative isopropyl group of Nd(OiPr)_3_ initiated the polymerization of Lac-OCA to form a reactive center, which triggered the polymerization of Lac-OCA. As shown in Scheme 1, this process produces PLA chains that contain an isopropyl group on one end and a hydroxyl group on the other end (lactic acid ester groups).

### 2.3. TGA and DSC analysis of PLAs

**Figure 2:**
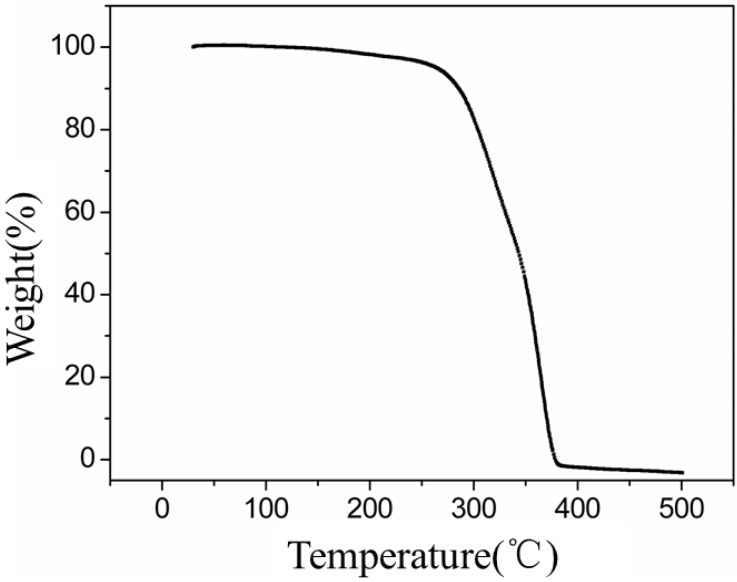
TGA curves of PLA (the PLA was synthesized in toluene-THF (toluene/THF = 2), Lac-OCA concentration = 1.67 mol/L, M/I = 150, temperature = 25 °C and reaction time = 4 h).

**Figure 3:**
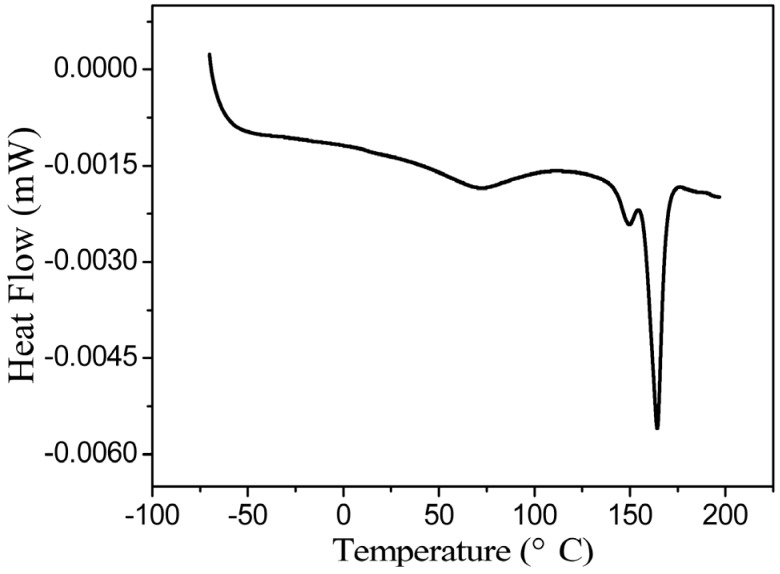
DSC curves of PLA (the PLA was synthesized in toluene-THF (toluene/THF = 2), Lac-OCA concentration = 1.67 mol/L, M/I = 150, temperature = 25 °C and reaction time = 4 h).

The PLAs were analyzed by TGA and DSC, and the TGA results are summarized in Figure 2. As shown in Figure 2, the initial decomposition temperature of PLA was 250 °C, which is slightly lower than that reported in the literature. Moreover, the temperature of complete decomposition was slightly higher than that reported in the literature. The DSC trace is shown in Figure 3. As illustrated in Figure 3, the glass transition temperature (Tg) and melting temperature of PLA occurred at 72 °C and 164 °C, respectively, which is consistent with the results reported in the literature.

## 3. Experimental

### 3.1. Materials

Triisopropoxyneodymium and aluminum triisopropoxide (Fluka Co., Basel, Switzerland) were dissolved in THF under nitrogen. Tetrahydrofuran (THF) and toluene were dried by refluxing over metallic sodium and distilling under a nitrogen atmosphere. Lithium 2-hydroxypropionate and triphosgene were purchased from Sichuan Biochem-ZX Co., Ltd. (Sichuan, China) and were used as received without further purification.

### 3.2. Synthesis of l-lactic Acid O-Carboxyanhydride (Lac-OCA)

Lac-OCA was prepared as described previously [9,10,11], however, triphosgene was used rather than diphosgene. Briefly, the lithium salt of l-lactic acid (0.5 mol) was suspended in anhydrous THF (350 mL) at 0 °C. Next, a solution containing triphosgene (0.3 mol) in anhydrous THF (150 mL) was added dropwise at 0–5 °C, and the reaction mixture was stirred at room temperature for 3 h. THF was removed under reduced pressure, and diethyl ether (1 L) was added to the residue. Lastly, the lithium salts were removed by filtration, and recrystallization from *tert*-butyl methyl ether afforded colorless crystals in 59% yield.

### 3.3. General Procedure for the Polymerization of Lac-OCA

Lac-OCA and solvent were added into a flame-dried glass reactor, which was purged with nitrogen several times prior to the addition. The initiator was dissolved in THF and was injected into the reactor using a syringe. The reaction was carried out at a predefined temperature for a suitable period of time. Upon completion, HCl (2 M) was added to the mixture to quench the reaction. The resulting mixture was diluted with toluene and washed twice with dilute HCl and water. The organic layer was concentrated to 1/3 of the original volume, and excess cold methyl alcohol was added to isolate the polymer. The purified product was dried under vacuum at 40 °C for 48 h.

### 3.4. Characterization Techniques

^1^H-NMR spectra of the polymers dissolved in CDCl_3_ were recorded using a Varian UNITY INOVA-400 MHz apparatus at 25 °C. The molecular weight and molecular distribution were determined by GPC using a Waters Associates ALC/GPC 244 apparatus at room temperature. For the GPC analysis, a differential refractometer was used as the detector, THF was employed as the solvent, and the instrument was calibrated using polystyrene standards. Differential scanning calorimetry (DSC) measurements of dried samples were performed from −70 to 200 °C at a heating rate of 10 °C/min on a TA Instrument (TA Q2000, New Castle, DE, USA). Thermal gravimetric analysis (TGA) was carried out using a thermal analyzer (STA 449, Netzsch, Selb, Germany). TGA analyses were performed at 40 °C to 500 °C at a rate of 10 °C/min in a nitrogen atmosphere.

## 4. Conclusions

In conclusion, Lac-OCA exhibited remarkable reactivity upon initiation by Nd(OiPr)_3_, and PLAs with controlled molecular weights and narrow polydispersities were obtained under mild conditions. NMR analysis showed that one end of the PLA chain contained an isopropyl group, while the other end contained a hydroxyl group (lactic acid ester groups). Thus, the functional groups of the initiator were chemically bonded to the polylactic acid chain. By changing the functional groups of the initiator, polylactic acids with different terminal functional groups can be obtained. Due to its availability and polymerizability, Lac-OCAs are promising monomers for the preparation of tailored architectures derived from well-defined PLAs.
